# Search trends and prediction of human brucellosis using Baidu index data from 2011 to 2018 in China

**DOI:** 10.1038/s41598-020-62517-7

**Published:** 2020-04-03

**Authors:** Chenhao Zhao, Yuhan Yang, Songyu Wu, Wenchao Wu, Hetian Xue, Kai An, Qing Zhen

**Affiliations:** 1 Department of Epidemiology and Biostatistics, School of Public Health, Jilin University, Changchun, China; 2 Key Laboratory of Zoonosis Research, Jilin University Department of Epidemiology and Biostatistics, School of Public Health, Changchun, 130021 China

**Keywords:** Bacterial infection, Epidemiology

## Abstract

Reporting on brucellosis, a relatively rare infectious disease caused by Brucella, is often delayed or incomplete in traditional disease surveillance systems in China. Internet search engine data related to brucellosis can provide an economical and efficient complement to a conventional surveillance system because people tend to seek brucellosis-related health information from Baidu, the largest search engine in China. In this study, brucellosis incidence data reported by the CDC of China and Baidu index data were gathered to evaluate the relationship between them. We applied an autoregressive integrated moving average (ARIMA) model and an ARIMA model with Baidu search index data as the external variable (ARIMAX) to predict the incidence of brucellosis. The two models based on brucellosis incidence data were then compared, and the ARIMAX model performed better in all the measurements we applied. Our results illustrate that Baidu index data can enhance the traditional surveillance system to monitor and predict brucellosis epidemics in China.

## Introduction

Human brucellosis is one of the most common zoonotic diseases, among many infectious diseases, including zoonoses^1^. After the initial infection, brucellosis has a wide range of clinical manifestations in the acute stage: patients can develop fever, sweating, and arthritis. Without appropriate treatment, the disease will progress to the chronic stage, which is far more severe than the acute stage^2^, such as endocarditis, neurological diseases^3,4^. Brucellosis is transferred from livestock to humans through nonsterile or contaminated products or contact with sick animals, and there have been no clear cases of brucellosis transfer from person to person^5^. Brucellosis not only endangers the development of the animal husbandry industry but also seriously threatens the health of the occupational population and people in epidemic areas. The health burden of brucellosis has caused severe damage to the health and economies of the affected countries. Thus, in the last few years, increasing attention has been paid to the epidemiology surveillance of brucellosis incidence^6^.

Currently, brucellosis appears in more than 160 countries and regions; the annual average number of new cases is higher than half a million globally^7^. In China, the incidence of brucellosis varies annually. There was no consistent pattern in incidence from 2011 to 2016; however, after 2016, there has been a distinct decrease. Other studies have also drawn the same conclusion either nationally or provincially^7–9^. The areas with brucellosis outbreak are mainly distributed in the northern provinces^10^. Moreover, brucellosis has several other epidemiological characteristics. For example, according to a previous study and reports, in terms of age and gender, young and middle-aged males in the labour force, especially those who work with livestock, are the main population being infected^3^.

The Internet has become a way for increasing numbers of people to acquire health information^11^. Internet data have entered the public health field with rapid updating and availability in large quantities. By analyzing Internet data, researchers can establish estimates of the distribution of health-related events in an Internet-based monitoring system model^12^. The *Autoregressive Integrated Moving Average* (ARIMA) model is a widely used model that can use the past and the present to predict the future^13^. The ARIMA model has been applied to predict other diseases epidemics such as AIDS^14^.

A well-known example of the use of Internet data in health is the monitoring of influenza outbreaks, where it is as accurate as traditional methods^11^. Google Trends is an Internet platform that specifies the term “public health surveillance” to provide users with geospatial and temporal patterns of search volume^15^. In the United States, researchers have successfully predicted flu outbreaks by analyzing the Google Trends and accurately estimated flu levels 1–2 weeks earlier than the published CDC report^16^. They also used Google Search to accurately predict influenza activity levels every week across the United States. Google Trends is widely used around the world, but it is not available in China. Most Chinese people use the Baidu engine to search for information on the Internet^17^. At present, Baidu, as the largest search engine on China’s Internet, has a vast share of the search engine market^18^. The information generated by the Baidu search data could be applied to epidemiological research. In Zhejiang Province of China, the prevalence of Norwalk virus has been determined through Internet monitoring (Baidu Index), and an appropriate model has been established to predict potential Norwalk virus infection^19^. Similarly, some methods of using Baidu Search Index(BSI) data have also been carried out in HIV/AIDS; hand, foot and mouth disease; and dengue^17,20,21^.

The current brucellosis surveillance system in China is flawed and incomplete. The reported number of human brucellosis cases may be only a portion of the actual cases of human brucellosis in the country^22^. Therefore, we need to adopt a more pragmatic approach to monitor the prevalence of human brucellosis. Based on previous studies of using search index data for the prediction of disease incidence, we believe that the Baidu index could provide a new approach to monitor the incidence of brucellosis.

## Results

### Descriptive and correlation analysis

**Figure 1:**
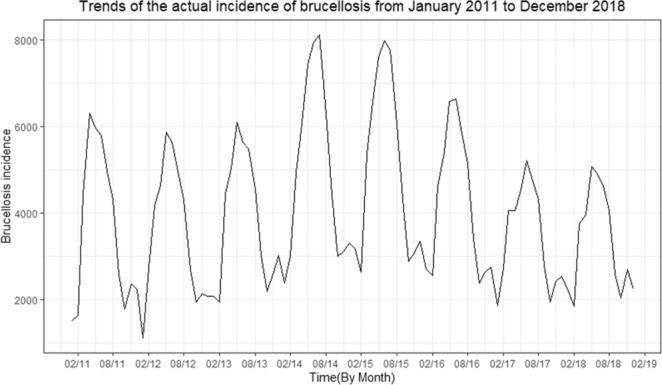
Trends of the actual incidence of brucellosis from January 2011 to December 2018.

The average incidence of human brucellosis from 2011 to 2018 is 48,111.63 per year. According to the time series plot of the human brucellosis incidence (Fig. 1). The annual incidence increased from 43,827 in 2011 to 60,782 in 2015; decreased from 2015 to 40,028 in 2018. The time distribution of the cases showed strong seasonality: mainly occurred from March to August, spring, and summer.

**Figure 2:**
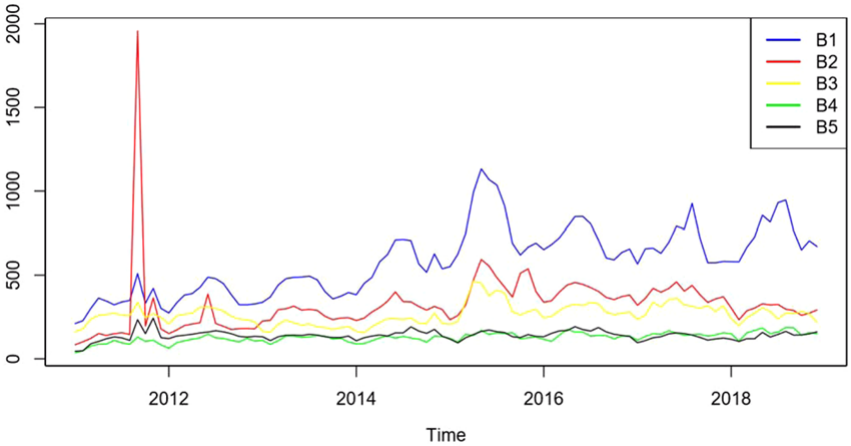
Time series of 5 different index terms from January 2011 to December 2018.

Table 1 is a brief description of the keyword data of BSI. The BSI data showed seasonal fluctuations and reached a peak in summer. We can infer from Figs. 1 and 2 that a similar seasonality occurred among cases of brucellosis and BSI data of keywords.

**Table 1:** Description of the observed data, there are five forms used to express brucellosis.

Abbr.	Min.	Mean	Median	Max	Std.Dev.
B1	211	582.5	580.5	1133	201.2
B2	84	324.9	305.5	1957	197.6
B3	155	264.2	261.5	465	60.5
B4	35	127.2	130.5	187	28.0
B5	45	140.0	140.0	243	28.3

B1, B2, B3, B4, and B5 are the different keywords describing brucellosis in the Chinese language.

Spearman correlation analysis was applied to find the correlation between the monthly observed brucellosis incidence and the BSI data (Table 2). The study showed a positive association between the BSI data and the monthly cases of brucellosis.

**Table 2:** The results of Spearman correlation analysis between BSI data and actual brucellosis incidence shows that all Baidu search indexes correlated with the time series of brucellosis incidence.

Abbr.	r_s_	P-value
B1	0.491	<0.01
B2	0.362	<0.01
B3	0.426	<0.01
B4	0.447	<0.01
B5	0.501	<0.01

We used cross-correlation analysis to further detect the correlation between the incidence of brucellosis and the lag order of BSI (Table 3). All keywords showed a significant relationship (p < 0.05). Two correlation analyses showed a significant relationship between the BSI data and the incidences of brucellosis, indicating the BSI data could reflect the changes in the brucellosis epidemic.

**Table 3:** The results of cross-correlation analysis between BSI data and actual brucellosis incidence. CCF: cross-correlation function.

Abbr.	Maximum CCF	Lag	P-value
B1	0.491	0	<0.01
B2	0.212	0	0.038
B3	0.476	0	<0.01
B4	0.367	0	<0.01
B5	0.391	0	<0.01

### Seasonal ARIMA model building and diagnosis

The seasonal ARIMA model uses past values of brucellosis incidence to predict future value. The result of the augmented Dickey-Fuller (ADF) test was not significant (Dickey-Fuller = -3.2995, lag order = 6, p = 0.076), suggesting that the time series is not stable and must be differentiated. After one-order differentiation, a method that could stationalize a non-stationary time series by computing the differences between two consecutive observations, the result of the ADF test is significant (Dickey-Fuller = -8.394, lag order = 6, p < 0.001). After differentiation, we use seasonal differentiation to eliminate the seasonality of the time series.

After all the differentiating, we use ACF(autocorrelation function) and PACF (partial autocorrelation function) plots of the time series in the supplement to estimate the MA and AR terms of the model (Supplementary Fig. S2). According to the minimum information quantity of the Akaike information criterion (AIC), after several trials, we selected ARIMA (1,1,1) (0,1,1)^12^ as the forecasting model (Table 4). We tested each coefficient of the parameter with the null hypothesis that the coefficient is zero. T in the table is the statistics of the t-test, and the p-value is the probability that the null hypothesis is correct. All the parameters are significant, and the residual autocorrelation test (Ljung-Box test) shows that the residuals are not distinguishable from a white noise series (Q* = 0.0057379, p = 0.9396).

**Table 4:** Parameters of ARIMA (1,1,1) (0,1,1)^12^.

	Coefficient	Standard error	T	P-value
AR1	0.606	0.169	3.576	<0.001
MA1	−0.817	1.150	−7.107	<0.001
SMA1	−0.762	0.209	−3.644	<0.001

### ARIMAX model construction and comparison

The ARIMAX model is a multivariable version of ARIMA. The BSI data are the covariables of the ARIMAX model. After several tries and external variable selection, we selected B1, B2, and B4 based on the minimal AIC values (Table 5), and we tested each coefficient the same way as the testing on the previous ARIMA model.

**Table 5:** The parameters of the ARIMA model with BSI as the external variable.

	Coefficient	Standard error	T	P-value
AR1	0.688	0.169	4.074	<0.001
MA1	−0.892	0.119	−7.524	<0.001
SMA1	−0.572	0.144	−3.972	<0.001
B1	3.057	0.815	3.751	<0.001
B2	−0.536	0.211	−2.530	0.011
B4	8.712	3.202	2.721	0.007

To evaluate the in-sample fitting performance of the models, we use ME (mean error), MAE (mean absolute error), MPE (mean percentage error), MAPE (mean absolute percentage error) RMSE (root mean square error), and AIC (Akaike information criterion) to compare models 1 and 2. Model 2 has better performance than model 1 in all measurements we applied. (Table 6)

**Table 6:** Comparison between the ARIMA and ARIMAX models.

	Model 1(ARIMA)	Model 2(ARIMAX)
ME	−16.3709	−3.4060
MAE	321.1026	292.1836
MPE	−0.7645	0.3039
MAPE	9.02%	8.07%
RMSE	445.6888	399.3604
AIC	1288.43	1259.64
Ljung-Box p	0.9396	0.7705

ME: mean error, MAE: mean average error, MPE: mean percentage error, MAPE: mean absolute percentage error, RMSE: root mean square error, AIC: akaike information criterion.

To show the performance of forecasting, we compared the prediction results with criterion interval from January 2019 to May 2019 of two models (Table 7). Model 2 still performs better than Model 1, since the RMSE for model 2 is 371.1875 and 377.6349 for model 1.

**Table 7:** Actual brucellosis incidence in 2019 and the out-of-sample prediction for ARIMA (Model 1) and ARIMAX (Model 2).

Time	Actual incidence	Model 1		Model 2	
		Value	95%CI	Value	95%CI
Jan 2019	2390	1696.793	(735.412, 2658.175)	1851.391	(1007.858, 2694.924)
Feb 2019	2227	1833.312	(609.734, 3056.889)	1738.133	(659.862, 2816.403)
Mar 2019	4021	3808.437	(2430.329, 5189.545)	4035.677	(2823.679, 5247.675)
Apr 2019	4559	4406.562	(2919.176, 5893.947)	4927.924	(3627.244, 6228.604)
May 2019	5238	5333.362	(3759.529, 6907.195)	5391.204	(4025.604, 6756.804)

## Discussion

This study used the keyword digging tool to find brucellosis-related keywords. We introduced different combinations of the keywords as covariables of the ARIMAX model. Since there are only five different keywords, we could compare models with all possible combinations. The fact that our selected model (model 2) has a lower AIC value than the model with all keywords combined (1259.64 VS 1278.95) is the reason we do not introduce the combination of the keywords into the model even though we calculated it.

Since Internet-based epidemiology related data is inexpensive, fine-grained, and real-time capable, it can fill the gap in public health supervision^23^. Therefore, in recent years, as an innovative way to improve the effectiveness of disease prevention and control programs, Internet-based monitoring systems are increasingly being explored^17^. Many similar studies have been conducted on various diseases. According to the World Health Organization (WHO), the actual incidence of brucellosis is 10 to 25 times higher than that reported^24^. The convenience of the Internet has attracted millions of people around the world to choose the Internet as a way to obtain health information^25^.

BSI shows the changes in the public in interest for time series in the selected term. In addition, different terms can be compared simultaneously. The source of the interest from the public about the brucellosis could be the news reports or the actual incidence. As a relatively rare disease, the news reports about the brucellosis are significantly less than common infectious diseases such as flu. The primary source of the online search queries could come from the brucellosis patients and the people around the patients. This hypothesis is the reason BSI could reflect the actual brucellosis incidence through the interests of the online search queries.

The ARIMA model is the most common model used to predict the epidemiological characteristics of a disease, but it can only make use of historical data. ARIMAX, however, can combine BSI and past incidence numbers to predict future incidence.

Additionally, in this study, Table 6 has shown that the ARIMAX model has a better fitting outcome than the ARIMA model. Thus, the BSI can play a vital role in establishing the ARIMAX model as well as the monitoring of brucellosis. The BSI of the previous month can be available at the beginning of each month. Moreover, through this model, the prediction of the cases of brucellosis can be calculated immediately, which is faster than the traditional monitoring system. When the value predicted by the model increases, it may indicate a rise in the incidence of brucellosis, and the relevant departments related to disease control and prevention can make preparations for potential outbreak brucellosis.

This study nevertheless has some deficiencies: First, China is a country with a wide range of cultures ethnicities, geographies, and population distributions^26^. Due to the scarcity of data at the provincial and municipal levels, it is not possible to predict the incidence trend in individual provinces and cities through this model. If data for each region can be collected, the forecast of brucellosis incidence can be targeted to different towns and areas. Second, according to the statistics of the China International Network Information Center, by December 2018, the Chinese Internet penetration rate was 59.6% (https://www.cnnic.net.cn/). This relatively low rate suggests that the Internet is not yet widely available in various remote parts of China. BSI cannot cover these areas. Therefore, the accuracy of predicting the number of cases with this model needs further evaluation. Third, although its exposure rate is relatively low compared to that of other diseases, it is still not possible to identify the reason for the search for brucellosis. Some queries may come from patients who are not sick and seek information for other purposes. When media reports on a new outbreak, the BSI will increase as a result of curiosity and panic, which leads to a false spike in the number of searches. Thus, further research is needed to find methods to eliminate the impact of media on the results^17^.

## Materials and Methods

### Data sources

Epidemiological data for brucellosis: We used the monthly reported data from the official website of the Chinese Center for Disease Control and Prevention (China CDC), which covers the period from January 2011 to December 2018. These data are aggregated, open to the public, and do not contain any personal information. We accessed the official website of the CDC (http://www.chinacdc.cn/), viewed the monthly report, and gathered the information of monthly brucellosis incidence into the table.

BSI data: We obtained monthly search engine query data from the Baidu index (https://index.baidu.com) by month, along with the monthly reported brucellosis incidence data. Because the BSI data were available from January 2011, we collected BSI data from January 2011 to December 2018.

Data from both sources were entered into Microsoft Excel 2016. The data were then exported in CSV format to R 3.6.1 for further analysis.

### Keyword selection

We used the keyword excavation tool (http://www.7c.com/keyword/) to excavate the different expressions that refer to brucellosis in the Chinese language. After removing the search terms that were not related to brucellosis from the excavation results, five different keywords that express the same meaning in the Chinese language were selected. Then, data for each keyword were collected separately from the Baidu Index website.

After the correlation analysis, a five-keywords-combined variable was calculated as follows:

1 $$weigh{t}_{i}=\frac{{\rho }_{i}}{{\sum }_{i=1}^{n}{\rho }_{i}}$$

2 $$combined\,BSI=\mathop{\sum }\limits_{i=1}^{n}weigh{t}_{i}\times keywor{d}_{i}$$

Where n is the number of keywords (n = 5), $keywor{d}_{i}$ and $weigh{t}_{i}$ were *i* th keywords value of BSI data and the weight of *i* th keyword. The ${\rho }_{i}$ are the value of Spearman correlation coefficients.

In the ARIMAX model constructing process, we tried a single keyword, the combination of several keywords or the combination of five keywords as covariable of the model multiple times, and we ultimately chose B1, B2, and B4 as the final covariables of the model based on the AIC criteria.

### ARIMA and ARIMAX

The ARIMA model is one of the most common time series models that use the historical value of a variable to predict its future values. The ARIMAX, multivariable version ARIMA, can take other time series information as the external variable.

We use both the ARIMA and ARIMAX models to predict the same time series, which is the incidence of brucellosis. The difference between the two models is that the second model (model 2) is multivariable.

Three steps were mainly introduced for model construction. Firstly, the autocorrelation term was determined through the ACF and PACF of brucellosis cases. Secondly, ARIMA and ARIMAX model was built. Finally, select the best performing model as the forecasting model.

### Model selection and prediction

We followed several criteria for time series model selection; first, the residuals of the models should not be distinguishable from a white noise series; second, when comparing two models, we assume the better model with lower absolute value in AIC and RMSE than the other model. After we find the best single-variable model and best multi-variable model, more measurements would be taken into the comparison between ARIMA and ARIMAX model, including ME, MAE, MPE, and MAPE.

Each of the measurements has its benefits. MPE is easy to calculate, and the result can be easily understood. MAPE is similar to MPE and can better represent the forecast error. MAE and RMSE are similar; the main difference between the two measurements is the sensitivity to the outlier of forecasting outcome; RMSE is more sensitive than MAE.

**Figure 3:**
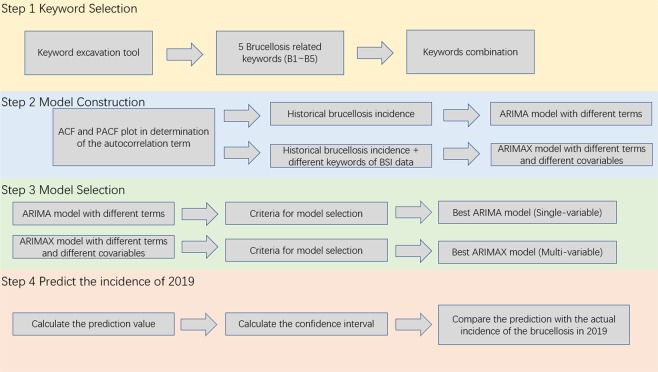
The whole research process from the selection of keywords to out-sample prediction.

After model selection, we predicted the future value from the selected model. We did five times one-month-ahead prediction and compared the actual brucellosis cases from January to May of 2019. The Fig. 3 briefly summarized the whole research process from the selection of keywords to out-sample prediction.

### Statistical tests

Spearman rank correlation was used to detect the association between monthly BSI data and brucellosis incidence.

Cross-correlation analysis is a measure of similarity between two time series. The range of results of this test is −1 to 1, and the closer the result to 1 or −1, the more closely two-time series are related.

The augmented Dickey-Fuller test is a unit root test to determine whether the differencing of a time series is required. In our analysis, the null hypothesis is that the data are stationary; a p-value less than 0.05 (p < 0.05) suggests that differencing is required.

The Ljung-Box test is the test that we applied to detect the autocorrelations of the residual series. If the p-value of the test is significant, we suggest that autocorrelations exist in the series, which indicates that the model does not fully utilize the information of the training set.

All statistical and modeling processes for the time series were analyzed using R 3.6.1. The ARIMA and ARIMAX model were built and analyzed using the “TSA” package. P < 0.05 was considered significant in the analysis process.

## Supplementary information

Supplementary Information.
