# Supplementary material for: Search trends and prediction of human brucellosis using Baidu index data from 2011 to 2018 in China
Source: Sci Rep. 2020 Apr 3;10:5896. doi: 10.1038/s41598-020-62517-7 (PMC7125199; doi:10.1038/s41598-020-62517-7)
Supplement: Supplementary file 1 — Supplementary Information. [file 41598_2020_62517_MOESM1_ESM.pdf]

Search trends and prediction of human brucellosis using Baidu index data from 2011 to 2018 in China

Chenhao Zhao[1], Yuhan Yang[1], Songyu Wu [1+], Wenchao Wu[1+], Hetian Xue[1+], Kai An[1], and Qing Zhen[*]

[1]Jilin University Department of Epidemiology and Biostatistics, School of Public Health,

[*] Key Laboratory of Zoonosis Research，Jilin University Department of Epidemiology and Biostatistics, School of Public Health, Changchun, 130021, China. zhenqing@jlu.edu.cn

[+]these authors contributed equally to this work

Supplementary Information

Abbr.	Description	Min.	Mean	Median	Max	Std.Dev.
**Bru**	Brucellosis actual incidence reported by Chinese CDC	1123	4009.3	4000	8102	1726.0
**B1**	布病	211	582.5	580.5	1133	201.2
**B2**	布氏杆菌病	84	324.9	305.5	1957	197.6
**B3**	布鲁氏菌病	155	264.2	261.5	465	60.5
**B4**	布鲁菌病	35	127.2	130.5	187	28.0
**B5**	布鲁氏杆菌病	45	140.0	140.0	243	28.3

Fig S1. Description of the observed data, there is five forms to express brucellosis the disease. Bru is the incidence of brucellosis. B1, B2, B3, B4, and B5 are the different keywords of brucellosis in the Chinese language. The keywords in Chinese language has shown in description.

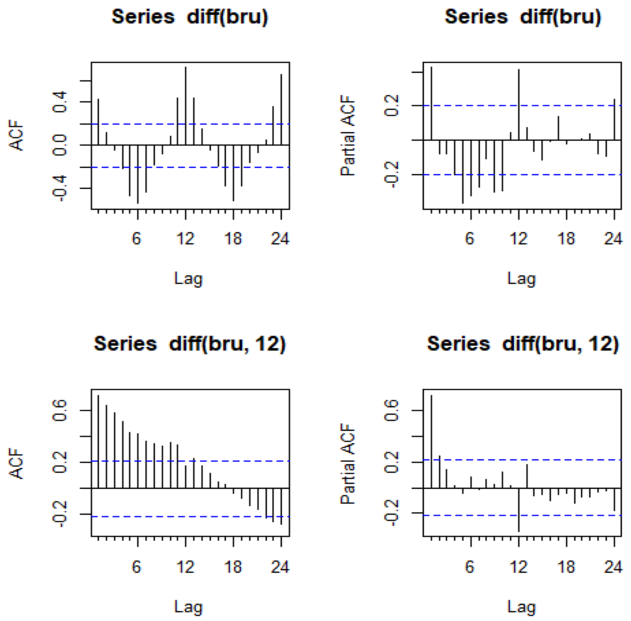

Fig S2. ACF and PACF plot of the one-order differentiated time series of brucellosis incidence and seasonal differentiated time series.
